# Roll of Members of the International Hahnemannian Association

**Published:** 1882-09

**Authors:** 


### Roll of Members of the International Hahnemannian Association.

| | | |
|---|---|---|
| 1880, | Allen, H. C., M. D., | Ann Arbor, Mich. |
| 1882, | Arrowsmith, W. Samuel, M. D., | Wateringbury, Kent Co., England. |
| 1880, | Baer, O. P., M. D., | Richmond, Ind. |
| " | Ballard, E. A., M. D., | Chicago, Ill. |
| 1881, | Bayard, Edward, M. D., | New York, N. Y. |
| " | Bedell, R. H., M. D., | Fremont, N. Y. |
| " | Bell, J. B., M. D., | Boston, Mass. |
| 1880, | Berridge, E. W., M. D., | London, England. |
| 1881, | Biegler, J. A., M. D., | Rochester, N. Y. |
| " | Birdsall, T. P., M. D., | Wappinger's Falls, N. Y. |
| " | Brown, Titus L., M. D., | Binghamton, N. Y. |
| " | Bruns, F., M. D., | Boston, Mass. |
| " | Butler, C. W., M. D., | Montclare, N. J. |
| " | Carleton, E., Jr., M. D., | New York, N. Y. |
| " | Clark, George H., M. D., | Philadelphia, Pa. |
| 1882, | Clausen, Daniel W., M. D., | Auburn, N. Y. |
| 1880, | Cranch, Edward, M. D., | Erie, Pa. |
| 1881, | Custis, J. B. Gregg, M. D., | Washington, D. C. |
| " | Dunn, J. L., M. D., | Titusville, Pa. |
| 1882, | Ehrman, Fred'k, M. D., | Cincinnati, Ohio. |
| " | Ehrman, Benj., M. D., | " " |
| 1881, | Fellger, Adolph, M. D., | Philadelphia, Pa. |
| 1880, | Foote, George F., M. D., | Stamford, Conn. |
| 1881, | Gale, Geo. Goldsworthy, M. D., | Quebec, Canada. |
| 1880, | Gentry, Wm. D., M. D., | Wyandotte, Kansas. |
| 1881, | Gregg, R. R., M. D., | Buffalo, N. Y. |
| " | Griffin, John F., M. D., | Williamsport, Pa. |
| " | Guernsey, Wm. Jefferson, M. D., | Philadelphia, Pa. |
| " | Hall, John, M. D., | Toronto, Canada. |
| " | Hatch, Horace, M. D., | Washington, D. C. |
| " | Haynes, J. R., M. D., | Indianapolis, Ind. |
| 1882, | Hunt, DeForest, M. D., | Grand Rapids, Mich. |
| 1881, | James, Walter M., M. D., | Philadelphia, Pa. |
| 1880, | Jenney, W. H., M. D., | Kansas City, Mo. |
| " | Kenyon, L. M., M. D., | Buffalo, N. Y. |
| 1881, | Kern, W. H., M. D., | McKeesport, Pa. |
| 1882, | Lawton, C. H., M. D., | Wilmington, Del. |
| 1881, | Lee, E. J., M. D., | Philadelphia, Pa. |
| 1880, | Leonard, W. H., M. D., | Minneapolis, Minn. |
| " | Lippe, Adolph, M. D., | Philadelphia, Pa. |

| | |
|---|---|
| 1881, Lippe, Constantine, M. D., | New York, N. Y. |
| " Miller, J. F., M. D., | Newark, N. J. |
| 1880, Mills, J. P., M. D., | Chicago, Ill. |
| 1881, Morgan, Laura, M. D., | San Francisco, Cal. |
| " Nash, Eugene B., M. D., | Cortland, N. Y., |
| " Nichols, C. F., M. D., | Boston, Mass. |
| 1880, Oliver, T. T., M. D., | Chicago, Ill. |
| 1881, Ostrom, H. J., M. D., | New York, N. Y. |
| " Payne, Fred'k W., M. D., | Boston, Mass. |
| 1882, Payne, Jas. H., M. D., | " " |
| 1880, Pearson, C., M. D., | Washington, D. C. |
| " Pomeroy, T. F., M. D., | Baltimore, Md. |
| 1881, Pompili, G., M. D., | Rome, Italy. |
| 1880, Poulson, T. Wilhelm, M. D., | Council Bluffs, Iowa. |
| 1882, Prentiss, C. E., M. D., | Washington, D. C. |
| 1881, Rendell, L. A., M. D., | New Haven, Conn. |
| " Roberts, John C., M. D., | New Utrecht, N. Y. |
| 1880, Rushmore, Edw., M. D., | Plainfield, N. J. |
| 1881, Schmitt, Julius, M. D., | Rochester, N. Y. |
| " Skinner, Thos., M. D., | Liverpool, England. |
| 1880, Smith, Thos. Franklin, M. D., | New York, N. Y. |
| 1881, Smith, Carleton, M. D., | Philadelphia, Pa. |
| " Swann, Samuel, M. D., | New York, N. Y. |
| 1880, Wells, P. P., M. D., | Brooklyn, N. Y. |
| 1881, Wells, L. B., M. D., | Utica, N. Y. |
| " Wesselhoeft, Wm. P., M. D., | Boston, Mass. |
| 1880, Wilson, T. P., M. D., | Ann Arbor, Mich. |
| " Winans, J. E., M. D., | Lyon's Farm, N. J. |
| " Wood, O. S., M. D., | Omaha, Neb. |

The following are elected, subject to compliance with the rules of the Association.

| | |
|---|---|
| 1882, Bradshaw, Wm., M. D., | Folkstone, Kent Co., England. |
| " Dunn, George, M. D., | London, England. |
| " Hoyne, T. S., M. D., | Chicago, Ill. |
| " Hussey, E. P., M. D., | Buffalo, N. Y. |
| " Mahoney, Edward, M. D., | Liverpool, England. |
| " Millspaugh, Chas. F., M. D., | Binghamton, N. Y. |
| " Walker, Arthur de Noe, M. D., | Ovington Gardens, England. |
| " White, Fred'k S., M. D., | Exeter, England. |

